# ﻿*Primulinaxingyiensis* (Gesneriaceae), a new species in the karst landforms of Guizhou Province, China

**DOI:** 10.3897/phytokeys.251.135126

**Published:** 2025-01-06

**Authors:** Jiang-Miao Gu, Song-Tao He, Fang Wen, Xin-Xiang Bai, Mei-Jun Li

**Affiliations:** 1 College of Forestry, Guizhou University, CN-550025, Guiyang, Guizhou, China; 2 Guangxi Key Laboratory of Plant Conservation and Restoration Ecology in Karst Terrain, Guangxi Institute of Botany, Guangxi Zhuang Autonomous Region and Chinese Academy of Sciences, CN-541006, Guilin, Guangxi Zhuang Autonomous Region, China; 3 National Gesneriaceae Germplasm Resources Bank of GXIB, Gesneriad Committee of China Wild Plant Conservation Association, Gesneriad Conservation Center of China (GCCC), Guangxi Institute of Botany, Botanical Garden, Guangxi Zhuang Autonomous Region and Chinese Academy of Sciences, CN-541006, Guilin, Guangxi Zhuang Autonomous Region, China

**Keywords:** Endemic species, Gesneriaceae, Karst, morphology, phylogeny, taxonomy

## Abstract

*Primulinaxingyiensis* X.X.Bai & F.Wen, a new species of Gesneriaceae in the karst landforms of Xingyi City, Guizhou Province, China, is described and illustrated. Morphologically, the species is similar to *P.davidioides* on corolla, while it is distinguished from *P.davidioides* by characteristics of thinner and smaller leaves, shorter peduncles, fewer flowers, smaller corolla, glabrous staminodes, and ovary shorter than style. Molecular phylogenetic analysis based on the combined dataset of *rpl*32-*trn*L, *trn*L-*trn*F, *atp*B-*rbc*L, and ITS sequences of the new species and 151 other species of *Primulina* Hance showed that the two populations of *P.xingyiensis* clustered into a clade, while it was most closely related to *P.malingheensis*. Following criteria D1 in the IUCN Red List Categories and Criteria, the new species should be assessed as ‘vulnerable’ (VU).

## ﻿Introduction

*Primulina* Hance is a genus in Gesneriaceae, and the morphology of species in this genus is complex, but variation in corolla is limited and usually infundibuliform (Wang et al. 2011; Weber et al. 2011; Möller et al. 2016). As of October 2024, a total of 257 taxa have been described in *Primulina*, of which 242 are distributed in China (GRC 2024; Wen et al. 2024). The number of taxa reported in this genus has increased in recent years, as well as the attention from more and more researchers and enthusiasts of Gesneriaceae. Most *Primulina* species are located in the south and southwest of China, which represent a classic example of karst landforms. Species of the genus have extremely high diversity in karst landforms, and most of the species are endemic and have a narrow distribution range (Gao et al. 2015; Xu et al. 2017; Wen et al. 2024). Qianxinan Buyi and Miao Autonomous Prefecture, Guizhou Province, is located in the southwest of China, adjacent to Yunnan Province and Guangxi Zhuang Autonomous Region, and is one of the most typical karst landforms in the world (Ye et al. 2020). So far, a total of more than 60 species of Gesneriaceae have been recorded in the region, of which six *Primulina* species, and new taxa of Gesneriaceae have been consistently reported in the region in recent years (Pan et al. 2021; Zhang et al. 2021; Huang et al. 2022; Zhou et al. 2023).

In April 2022, during a survey of rock plants in karst regions in Xingyi City, Qianxinan Buyi and Miao Autonomous Prefecture, China, our research group discovered a *Primulina* species that was not in flower and was growing on the rock walls in the weak light area of a karst cave in Jingnan Town. In July of the same year, we went to the site again to survey, and these plants were in flower, with conspicuous large bracts and purple corollas. In July of the following year, we discovered another population of the species in a karst cave in the Wanfenglin National scenic spot in Xingyi City, which is less than 10 kilometers in a straight line from Jingnan Town. After close examination of the morphological characteristics of the species, we found it to be clearly different from any of the formally reported species of *Primulina*. Even though the corolla morphology of the species is similar to that of *P.davidioides* F.Wen & Xin Hong (Hong et al. 2018), the characteristics of the size of leaves, the length of peduncles, the number of flowers, the size of the corolla, the glabrous staminodes, and the length ratio of style to ovary are distinguished from *P.davidioides*. Therefore, we confirmed that the species is an undescribed species of *Primulina* and be named *P.xingyiensis*, described and illustrated as a new species in this study. Moreover, molecular phylogenetic analysis based on the combined dataset of the chloroplast DNA sequences (*rpl*32-*trn*L, *trn*L-*trn*F, and *atp*B-*rbc*L) and the nuclear ribosomal internal transcribed spacers (ITS) sequences of the new species and 151 other species of *Primulina* confirmed the position of the species in *Primulina*.

## ﻿Materials and methods

### ﻿Morphological comparisons

Detailed anatomical photographs and pressed specimens of the species were taken in the field, and the morphological characteristics of more than 30 individuals were observed and recorded carefully. In conjunction with previous research, the plant was described following the terminology used by Wang and Pan (1990), Li and Wang (2005), and Weber et al. (2011). Morphological characters of the species were compared with the digitized type specimens of related species in herbaria P (https://science.mnhn.fr/all/search), PE (https://pe.ibcas.ac.cn/index.html), IBK (http://www.cfh.ac.cn/subsite/Albums.aspx?siteid=IBK), and GXMI (http://www.gicmp.com/) and with morphological descriptions in the primary literature.

### ﻿Taxon sampling

Mature leaves of the new species and *Primulinamalingheensis* X.X.Bai, F.Wen & Y.L.Zhou were collected from living plants free of pests and diseases from the type locality, and the Guangxi Institute of Botany provided mature leaves taken from the type locality of *P.davidioides*. These leaves were rapidly dried in silica gel. In addition, the *rpl*32-*trn*L, *trn*L-*trn*F, *atp*B-*rbc*L, and ITS sequences for 151 *Primulina* species and two *Petrocodon* species were downloaded from Genbank (Suppl. material 1). Two *Petrocodon* species were treated as outgroup.

### ﻿DNA extraction, PCR, and sequencing

Total DNA was extracted from silica-gel dried leaves of *Primulinamalingheensis*, *P.davidioides*, and both populations of the new species, respectively, using the Plant DNA Extraction Kit (Cat. No. B518261, Sangon Biotech, Shanghai, China). The nuclear ribosomal internal transcribed spacer (ITS) and chloroplast DNA sequences (*rpl*32-*trn*L, *trn*L-*trn*F, and *atp*B-*rbc*L) of these samples were amplified by polymerase chain reaction (PCR), using the primers in the research of Ning (2017), Taberlet et al. (1991), Mayer et al. (2003), Savolainen et al. (1994), and White et al. (1990) (Table 1). All DNA samples were sent to Sangon Biotech Co. Ltd. (Shanghai, China) for sequencing and splicing.

**Table 1.** Primers for amplification and sequencing.

DNA sequences	Primer	Sequence	Reference
*rpl*32-*trn*L	rpl32_58F	5’-GGTATTGTGCATCGTTAAAAGC-3’	Ning 2017
	trnL_58R	5’-GCTTCCTAAGAGCAGCGTGT-3’	
*trn*L-*trn*F	c	5’-CGAAATCGGTAGACGCTACG-3’	Taberlet et al. 1991
	f	5’-ATTTGAACTGGTGACACGAG-3’	
*atp*B-*rbc*L	JF31	5’-TTTCAAGCGTGGAAACCCCAG-3’	Mayer et al. 2003
	JF5	5’-TACAGTTGTCCATGTACCAG-3’	Savolainen et al. 1994
ITS	ITS1	5’-TCCGTAGGTGAACCTGCGG-3’	White et al. 1990
	ITS4	5’-TCCTCCGCTTATTGATATGC-3’	

### ﻿Phylogenetic analysis

The *rpl*32-*trn*L, *trn*L-*trn*F, *atp*B-*rbc*L, and ITS sequences were aligned separately using MAFFT version 7 (https://mafft.cbrc.jp/alignment/server/) (Katoh and Standley 2013), and the missing parts of the sequences at both ends were removed in MEGA_11.0.13 (Tamura et al. 2021) and adjusted manually. The *rpl*32-*trn*L, *trn*L-*trn*F, *atp*B-*rbc*L, and ITS sequences were concatenated in series in the Concatenate Sequence module of PhyloSuite_v1.2.3 (Zhang et al. 2020) and determined the optimal base substitution models for four partitions of the combined dataset in the PartitionFinder2 module using the corrected Akaike Information Criterion (AICc). Phylogenetic analysis of the combined dataset of *rpl*32-*trn*L, *trn*L-*trn*F, *atp*B-*rbc*L, and ITS sequences using Maximum likelihood (ML) and Bayesian inference (BI) in Phylosuite v.1.2.3. ML performed 5,000 ultrafast bootstrap searches (BS) to assess branch support. BI was performed for at least 10 million generations, sampled every 1,000 generations, and posterior probabilities (PP) were calculated to assess branch reliability. The number of conserved sites, variable sites, and parsimony-informative sites for the combined dataset was obtained via MEGA_11.0.13.

## ﻿Results

### ﻿Morphological comparisons

The species of *Primulina*, Tribe Trichosporeae, Subfam. Didymocarpoideae, Gesneriaceae, have large, striking bracts. Of the officially published species of the genus, *Primulinaeburnea* (Hance) Yin Z.Wang, *P.lunglinensis* (W.T.Wang) Mich.Möller & A.Weber, *P.davidioides*, *P.grandibracteata* (J.M.Li & Mich.Möller) Mich.Möller & A.Weber, *P.lungzhouensis* (W.T.Wang) Mich.Möller & A.Weber, etc., also have large bracts, of which *P.davidioides* are most similar to the species, but all two can still be distinguished morphologically. The new species is distinguished from *P.davidioides* by characteristics of thinner and smaller leaves, shorter peduncles, fewer flowers, smaller corolla, glabrous staminodes, and ovary shorter than style (Table 2).

**Table 2.** Morphological comparison of *Primulinaxingyiensis*, *P.malingheensis* and *P.davidioides*.

Characteristics	*P.xingyiensis*	*P.malingheensis*	*P.davidioides*
Leaf	Thin, slightly fleshy, 4.3–9.8 × 3.1–5.1 cm	Thickly chartaceous, more or less fleshy, 1.7–5.8 × 1.2–3 cm	Pachyphyllous, rigid and coriaceous when dry, (5–)11–12.5 × 5–10 cm
Peduncles	0.5–4 cm long	1.2–3.0 cm long	5–9(–12) cm long
Number of flowers	1–6 flowers per cymes	1–3 flowered per cymes	5–9(–11) flowers per cymes
Bracts	White when flowering, apex margin light green, occasionally purplish-red, narrowly ovate to suborbicular, 2.0–4.3 × 1.5–2 cm	Pale green to brownish-purple, ovate, 1.3–1.8 × 0.8–1.0 cm	White when flowering, cordate to suborbicular, 4–6 × 4–5 cm
Calyx	Lobes linear-lanceolate to lanceolate, white	Lobes lanceolate, light purple about 1/2 way down from top, yellowish green below	Lobes triangular, white to subtranslucent
Corolla	Purple, 4.98–5.26 cm long, a dark purple spot between adaxial lip lobes	Outside white, lips purple inside, 4.8–6.0 cm long, throat roof with a reddish-brown spot	Purple, ca. 6 cm long, throat roof with a dark purple spot
Anthers	Pale yellow	White, ventral surface slightly bluish purple	Pale yellow
Staminodes	Glabrous, central one ca. 1 mm long	Glabrous, central one ca. 1 mm long	Sparsely glandular-puberulous, central one ca. 4.5 mm long
Pistil	Pistil 3.58–3.65 cm, ovary 1.54–1.61 cm long, style ca. 2.0 cm long	Pistil ca. 3.8 cm long, ovary ca. 1.5 cm long, style ca. 2 cm long	Pistil ca. 3.7 cm long, ovary ca. 2.0 cm long

### ﻿Phylogenetic analysis

The combined dataset included 4115 characters (including 943 characters of ITS, 1177 characters of *atp*B-*rbc*L, 1119 characters of *rpl*32-*trn*L, and 876 characters of *trn*L-*trn*F), of which 2481 (60.29%) conserved sites, 1200 (19.16%) variable sites, and 780 (18.76%) parsimony-informative sites (Table 3). The optimal base substitution models for *rpl*32-*trn*L, *trn*L-*trn*F, *atp*B-*rbc*L, and ITS partitions obtained by PhyloSuite_v1.2.3 were GTR + I + G, GTR + G, GTR + I + G, and SYM + I + G, respectively (Table 3).

**Table 3.** Basic characteristics of the molecular dataset used in this study.

Sequences	*rpl*32-*trn*L	*trn*L-*trn*F	*atp*B-*rbc*L	ITS	Combined dataset
Number of sequences (ingroup/outgroup)	154/2	154/2	154/2	154/2	154/2
Aligned length (bp)	1119	876	1177	943	4115
Conserved sites (bp)	831	626	629	395	2481
Variable sites (bp)	229	190	303	478	1200
Parsimony-informative sites (bp)	134	95	183	368	780
Optimal base substitution models for four partitions	GTR + I + G	GTR + G	GTR + I + G	SYM + I + G	

**Figure 1.**
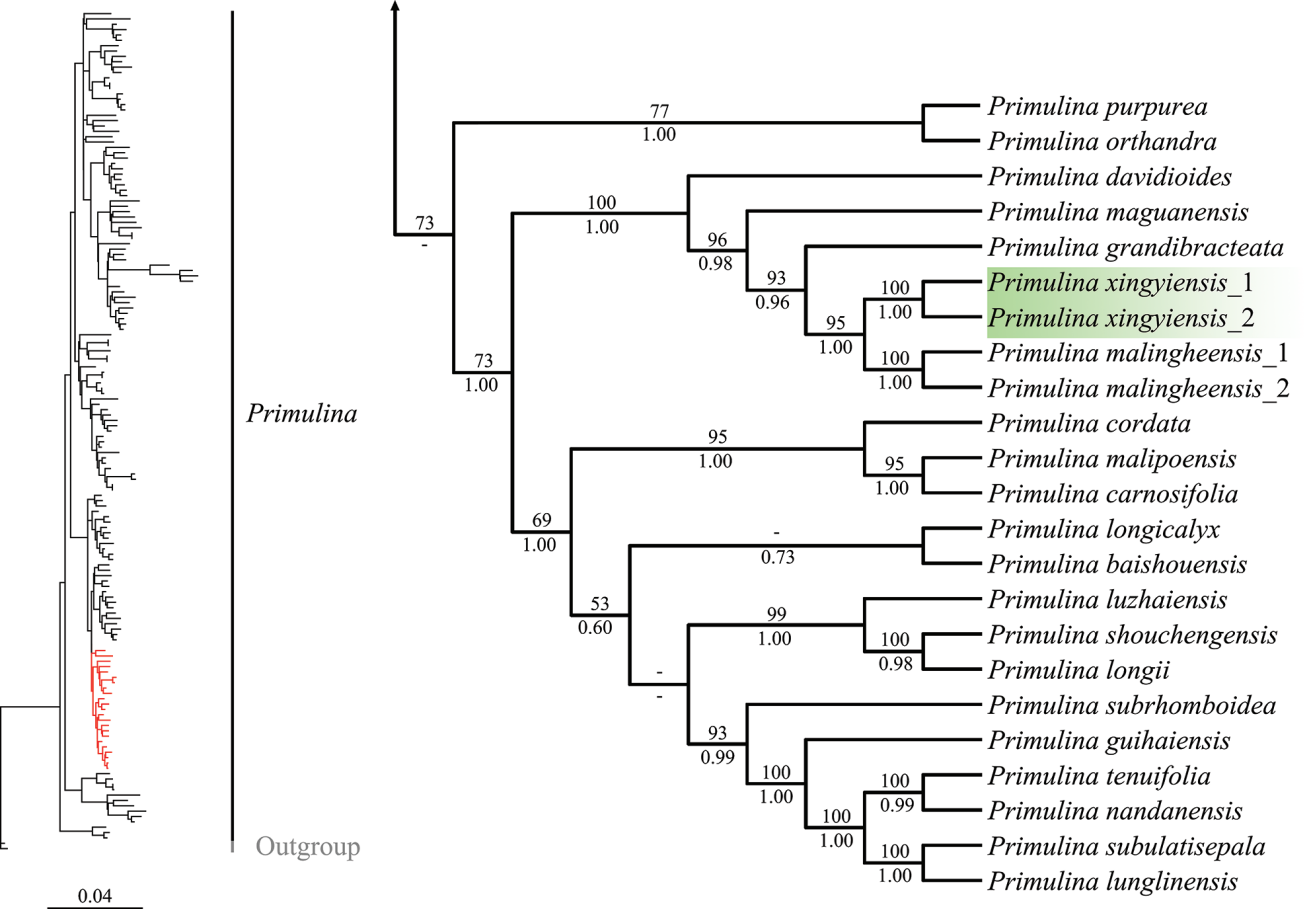
The partial ML and BI phylogenetic trees (red part) from the analyses of the combined dataset of the *rpl*32-*trn*L, *trn*L-*trn*F, *atp*B-*rbc*L, and ITS sequences. Numbers above branches are ML ultrafast bootstrap values; numbers below branches are BI posterior probability values. The green background indicates two populations of the new species.

ML and BI trees showed that the two populations of *Primulinaxingyiensis* formed a fully supported clade (BS = 100, PP = 1.00) sister to *P.malingheensis* (BS = 95, PP = 1.00), and *P.grandibracteata* is sister to the clade formed by *P.xingyiensis* and *P.malingheensis* (BS = 93, PP = 0.96); *P.davidioides* is not closely related to them (Fig. 1, Suppl. material 2).

### ﻿Taxonomic treatment

#### ﻿ Primulina xingyiensis

Taxon classificationPlantaeLamialesGesneriaceae

X.X.Bai & F.Wen sp. nov.

E9622A3C-B432-5D7C-AB38-7420B926763C

urn:lsid:ipni.org:names:77354654-1

Figs 2 , 3

##### Type.

CHINA • Guizhou: Xingyi City, Jingnan Town, growing on rock walls in limestone caves, 24°56'N, 104°53'E, elev. ca. 877 m, 6 July 2022, *X. X. Bai* et al. *XYS 07277* (holotype: GZAC!; isotype: IBK!).

##### Diagnosis.

The corolla morphology of *Primulinaxingyiensis* is similar to that of *P.davidioides*, and the phylogenetic tree shows that its closest relative is *P.malingheensis*, but there are also clear differences in morphological characteristics between the three (Table 2, Fig. 4). *Primulinaxingyiensis* can be distinguished from *P.davidioides* by the thinner and smaller leaves, slightly fleshy (vs. pachyphyllous, rigid and coriaceous when dry); the shorter peduncles, 0.5–4 cm long only [vs. 5–9(–12) cm long]; the fewer flowers, 1–6 flowers per cymes (vs. 5–9(–11) flowers per cymes); the smaller corolla, ca. 5 cm long (vs. ca. 6 cm long); the staminodes glabrous, central one ca. 1 mm long (vs. sparsely glandular-puberulous, central one ca. 4.5 mm long); the ovary shorter than style (vs. the ovary longer than style). *Primulinaxingyiensis* can be distinguished from *P.malingheensis* by the larger leaves, 4.3–9.8 × 3.1–5.1 cm (vs. 1.7–5.8 × 1.2–3 cm); the larger bracts, 2.0–4.3 × 1.5–2 cm, white when flowering, apex margin light green, occasionally purplish-red (vs. 1.3–1.8 × 0.8–1.0 cm, pale green to brownish-purple); the white calyx lobes (vs. light purple about 1/2 way down from top, yellowish green below); a dark purple spot between adaxial lip lobes (vs. throat roof with a reddish-brown spot); the pale yellow anthers (vs. white anthers, ventral surface slightly bluish purple).

**Figure 2.**
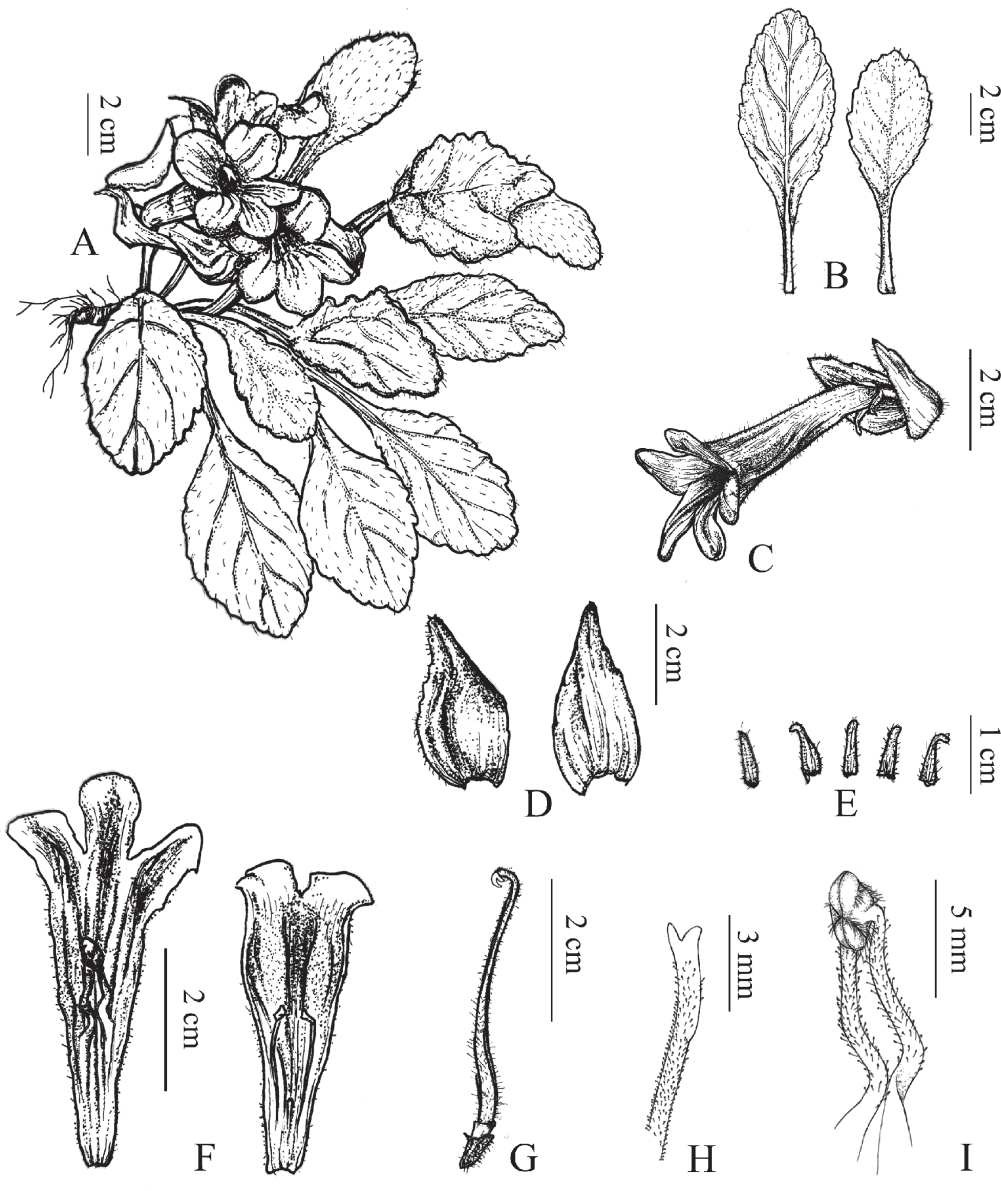
*Primulinaxingyiensis***A** plant **B** leaves **C** corolla **D** bracts **E** calyx lobes **F** corolla opened showing internal features **G** pistil and disc **H** stigma **I** stamens (Drawn by Bai-Qiu He).

**Figure 3.**
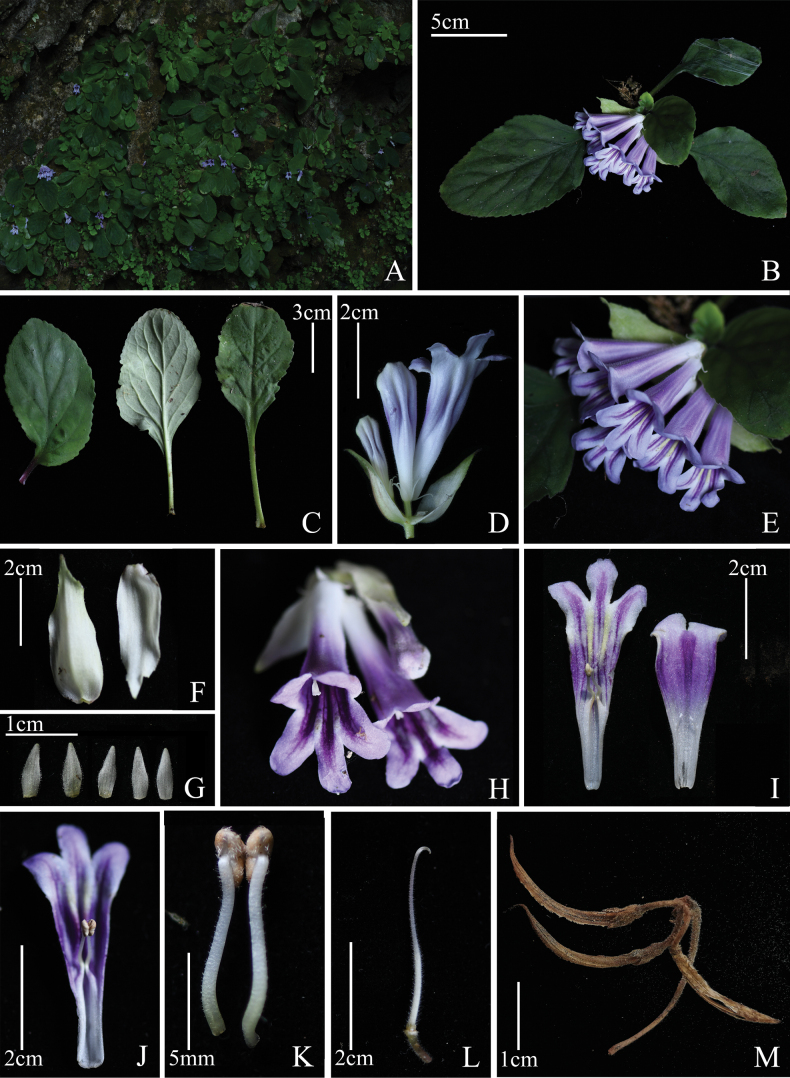
*Primulinaxingyiensis***A** habitat **B** plant **C** leaves **D, E** inflorescence **F** bracts **G** calyx lobes **H** front view of the corolla **I** corolla opened showing internal features **J** anthers **K** stamens **L** pistil and disc **M** fruits (Photographed by Xin-Xiang Bai).

**Figure 4.**
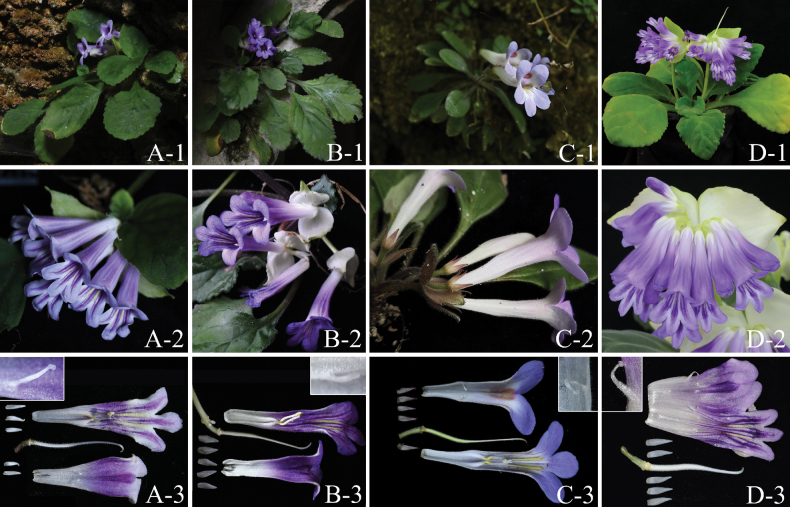
*Primulinaxingyiensis*, *P.malingheensis* and *P.davidioides***A***P.xingyiensis* of Jingnan Town **B***P.xingyiensis* of Wanfenglin **C***P.malingheensis***D***P.davidioides***A-1, B-1, C-1, D-1** Plant with flowering **A-2, B-2, C-2, D-2** Cymes **A-3, B-3, C-3, D-3** Floral anatomy, staminode indumentum (inset) (D-1 provided by Dr. Fang Wen; D-2 provided by Mr. Tao-Ran Chen; D-3 provided by Mr. De-Chang Meng).

##### Description.

Perennial herb. Rhizome subcylindrical, 5–16 mm long, 1.3–2 mm in diameter. Leaves all basal, 4 to 12, opposite; petiole 0.5–3.8 cm long, 1–3 mm in diameter, densely white pubescent; leaf blade slightly fleshy, ovate or oblong, (1.3–)4.3–9.8 × (0.7–)3.1–5.1 cm, margin irregular shallow dentate, puberulent on both sides, base cuneate, apex obtuse or suborbicular; midrib adaxially slightly impressed, abaxially prominent, lateral veins 2–5 on each side. Cymes 1–3 on each plant, 1–6 flowers per cymes; peduncle 0.5–4 cm long, ca. 2 mm in diameter, densely white pubescent. Bracts 2, opposite, narrowly ovate to suborbicular, white when flowering, apex margin light green, occasionally purplish-red, 2.0–4.3 × 1.5–2 cm, outside densely pubescent, inside sparsely pubescent, margin serrate from above the middle, base slightly truncate, apex acuminate; pedicels 5–9 mm long, ca. 1.5 mm in diameter, densely covered by short glandular hairs; calyx 5-lobed to base, lobes nearly equal in length, white, linear-lanceolate to lanceolate, 5–8 mm × 1.5–2.2 mm, outside with short glandular hairs, inside glabrous, margin entire, apex acuminate. Corolla purple, 4.98–5.26 cm long, outside with glandular hairs, inside with vertical purple stripes, sparsely short glandular hairs below the insertion of the filaments; tube infundibuliform, 3.65–3.82 cm long, 1.14–1.27 cm diameter at mouth, 4.7–5.1 mm in diameter at base; limb distinctly 2-lobed, adaxial lip 2-parted to near base, dark purple spot between lobes, lobes semi-elliptic, ca. 6 × 6 mm; abaxial lip ca. 1.8 cm long, 3-lobed to near middle, lobes oblong, 8–10 × ca. 6 mm. Stamens 2, inserted at 2.3 cm from the base of the corolla; filaments linear, 0.96–1.12 cm long, sparsely covered by short glandular hairs, distinctly geniculate, white, pale yellow basally or below the middle; anthers 2.9–3.2 mm long, 1.44–1.56 mm across, light yellow, reniform, fused by their adaxial surfaces, abaxial surfaces sparsely white bearded. Staminodes 3, glabrous, white, lateral ones ca. 4 mm long, inserted at 1.5 cm from the base of the corolla, central one ca. 1 mm long, inserted at 9 mm from the base of the corolla. Disc ca. 1 mm high, light yellow, annular, glabrous. Pistil 3.58–3.65 cm long, white, densely covered by white short glandular hairs, ovary linear, 1.54–1.61 cm long, 1.47–1.51 mm in diameter, densely covered by short glandular hairs; style linear, ca. 2 cm long, stigma 4.92–5.04 mm long, only with abaxial lip, oblique trapeziform, apex 2-lobed, lobes triangular. Capsule linear, 2.4–3.3 cm long.

##### Phenology.

Flowering from June to July and fruiting from August to November.

##### Etymology.

The specific epithet ‘*xinyiensis*’ refers to the type locality Xingyi. Its Chinese name is Xīng Yì Bào Chūn Jù Tái (兴义报春苣苔).

##### Distribution and habitat.

This new species is currently distributed only in the type locality of Xingyi City, Guizhou Province, China, where two populations have been found, both growing on limestone cave walls. Accompanying species mainly include *Petrocosmea* sp., *Adiantumcapillus-veneris* L., and *Aleuritopterisanceps* (Blanf.) Panigrahi.

##### Conservation status.

Currently, only two populations of *Primulinaxingyiensis* have been found in Xingyi City, Guizhou Province, with a straight-line distance of less than 10 km. The number of mature individuals in the two populations is about 500, and they are close to human settlements, making them susceptible to anthropogenic disturbances. We have not found the species again in similar habitats during extensive investigations in the adjacent areas. Since the estimated number of mature individuals is about 500, the new species should be assessed as ‘vulnerable’ (VU) according to criterion D1 of the IUCN Red List Categories and Criteria (IUCN 2022).

##### Additional specimens examined.

*Primulinamalingheensis*, China • Guizhou Province, Xingyi City, Maling River, 25°3'N, 104°59'E, ca. 877 m a.s.l., 18 May 2022, *X.X. Bai & Y.L. Zhou MLHXG202205187* (holotype: GZAC!).

##### Notes.

The sequences of *Primulinamalingheensis*_1 used in this study were uploaded into the GenBank database and labeled as *P.secundiflora* (Chun) Mich.Möller & A.Weber by Kong et al. (2017). At that time, researchers generally identified a plant collected in Xingyi City, Guizhou Province, China, as *P.secundiflora*. Later, our research team conducted a field survey of this species and found that it was significantly different from *P.secundiflora* and from other formally published species of *Primulina*, and we published it as a new taxonomic unit and named it *P.malingheensis* (Zhou et al. 2023). In this study, we obtained the sequences of the type locality of *P.malingheensis*, labeled as *P.malingheensis*_2. *Primulinamalingheensis*_1 and *P.malingheensis*_2 formed a fully supported clade in the phylogenetic tree (Fig. 1), and the *rpl*32-*trn*L, *trn*L-*trn*F, *atp*B-*rbc*L, and ITS sequences of *P.malingheensis*_1 and *P.malingheensis*_2 were only present at two, zero, one, and six variant sites. The sequences of *P.secundiflora* uploaded by Kong et al. (2017) actually misidentified *P.malingheensis* as *P.secundiflora*, so it is labeled as *P.malingheensis*_1 in this study.

## Supplementary Material

XML Treatment for Primulina xingyiensis

## ﻿Additional information

### Author ORCIDs

Jiang-Miao Gu https://orcid.org/0009-0004-4948-3944

Fang Wen https://orcid.org/0000-0002-3889-8835

Xin-Xiang Bai https://orcid.org/0000-0003-2449-6664

### Data availability

All of the data that support the findings of this study are available in the main text or Supplementary Information. The new data generated in the study were uploaded to the NCBI database (https://www.ncbi.nlm.nih.gov/) under the accession numbers PQ196587–PQ196590 and PQ201180–PQ201191.

## Supplementary materials

Supplementary material 1 Species information for constructing phylogenetic trees Data type xlsx https://binary.pensoft.net/file/1214482This dataset is made available under the Open Database License (http://opendatacommons.org/licenses/odbl/1.0/). The Open Database License (ODbL) is a license agreement intended to allow users to freely share, modify, and use this Dataset while maintaining this same freedom for others, provided that the original source and author(s) are credited.Jiang-Miao Gu, Song-Tao He, Fang Wen, Xin-Xiang Bai, Mei-Jun Li

Supplementary material 2 The ML and BI phylogenetic trees from the analyses of the combined dataset of the *rpl*32-*trn*L, *trn*L-*trn*F, *atp*B-*rbc*L, and ITS sequences Data type tif https://binary.pensoft.net/file/1214483This dataset is made available under the Open Database License (http://opendatacommons.org/licenses/odbl/1.0/). The Open Database License (ODbL) is a license agreement intended to allow users to freely share, modify, and use this Dataset while maintaining this same freedom for others, provided that the original source and author(s) are credited.Jiang-Miao Gu, Song-Tao He, Fang Wen, Xin-Xiang Bai, Mei-Jun Li
